# Teens Taking Charge: A Randomized Controlled Trial of a Web-Based Self-Management Program With Telephone Support for Adolescents With Juvenile Idiopathic Arthritis

**DOI:** 10.2196/16234

**Published:** 2020-07-29

**Authors:** Jennifer N Stinson, Chitra Lalloo, Amos S Hundert, Sarah Campillo, Tania Cellucci, Paul Dancey, Ciaran Duffy, Janet Ellsworth, Brian M Feldman, Adam M Huber, Nicole Johnson, Geert't Jong, Kiem Oen, Alan M Rosenberg, Natalie J Shiff, Lynn Spiegel, Shirley M L Tse, Lori Tucker, Joseph Charles Victor

**Affiliations:** 1 The Hospital for Sick Children Toronto, ON Canada; 2 Institute of Health Policy, Management, and Evaluation University of Toronto Toronto, ON Canada; 3 Montreal Children's Hospital Montreal, QC Canada; 4 Department of Pediatrics, Division of Rheumatology McMaster University Hamilton, ON Canada; 5 Department of Pediatrics, Division of Rheumatology Memorial University of Newfoundland St. John's, NL Canada; 6 Department of Pediatrics, Division of Rheumatology McGill Research Institute Montreal, QC Canada; 7 Department of Pediatrics, Division of Rheumatology University of Alberta Edmonton, AB Canada; 8 IWK Health Centre Halifax, NS Canada; 9 Alberta Children's Hospital Calgary, AB Canada; 10 Children's Hospital Research Institute of Manitoba Winnipeg, MB Canada; 11 Pediatrics and Child Health University of Manitoba Winnipeg, MB Canada; 12 University of Saskatchewan Saskatoon, SK Canada; 13 Biobehavioral Nursing Science University of Florida Gainesville, FL United States; 14 Hospital for Sick Children Toronto, ON Canada; 15 Department of Pediatrics, Division of Rheumatology University of British Columbia Vancouver, ON Canada; 16 Institute for Clinical Evaluative Sciences Toronto, ON Canada

**Keywords:** eHealth, randomized controlled trial, adolescents, juvenile idiopathic arthritis, self-management, self-efficacy, technology, patient education, internet, pediatric pain

## Abstract

**Background:**

Juvenile idiopathic arthritis (JIA) is a serious and potentially debilitating pediatric illness. Improved disease self-management may help to improve health outcomes.

**Objective:**

This study aimed to evaluate the effectiveness of the *Teens Taking Charge* Web-based self-management intervention in reducing symptoms and improving health-related quality of life (HRQL) in adolescents with JIA compared with a Web-based education control condition.

**Methods:**

Adolescents with JIA aged 12 to 18 years were recruited from 11 Canadian pediatric rheumatology centers. Caregivers were invited to participate along with their child. In addition to standard medical care, participants were randomized to receive either (1) the *Teens Taking Charge* self-management intervention or (2) a Web-based education control condition for a period of 12 weeks. Adolescents in the intervention group completed website modules addressing cognitive behavioral coping skills, stress management, and other self-management topics, while also receiving monthly telephone calls from a trained health coach. Adolescents in the education control group were instructed to view a series of preselected public JIA educational websites and received monthly calls from a coach who asked about *their own best efforts* at managing JIA. Caregivers in the intervention group completed website modules related to promoting independence and disease self-management in their child. Caregivers in the education control group were instructed to view a series of preselected public JIA educational websites. Outcome assessment occurred at baseline, 12 weeks (posttreatment), and at 6 and 12 months postrandomization. The primary outcomes were pain intensity, pain interference, and HRQL. Secondary outcomes were emotional symptoms, adherence, coping, knowledge, and self-efficacy.

**Results:**

In total, 333 adolescents and 306 caregivers were enrolled. Significant overall reductions in pain intensity (*P*=.02) and pain interference (*P*=.007) were observed for intervention group participants compared with those in the education control group, after adjusting for baseline levels. There was a significant overall improvement in HRQL related to problems with pain (*P*=.02) and problems with daily activities (*P*=.01). There was also a significant difference in the intervention group over time (*P*=.008) for HRQL related to treatment problems, with the intervention group participants demonstrating improved HRQL by 12 months compared with education control group participants. Both groups showed nonsignificant improvements compared with baseline in other primary outcomes. There were no significant differences between the groups in any secondary outcomes or caregiver-reported outcomes.

**Conclusions:**

The results of this randomized trial suggest that the *Teens Taking Charge* Web-based intervention is effective at reducing both pain intensity and pain interference, as well as improving HRQL in adolescents with JIA, compared with education control. These effects are sustained for up to 12 months following program completion. The *Teens Taking Charge* program is now publicly available at no cost.

**Trial Registration:**

ClinicalTrials.gov NCT01572896; https://clinicaltrials.gov/ct2/show/NCT01572896

## Introduction

### Background

Juvenile idiopathic arthritis (JIA) is a serious and potentially debilitating pediatric illness. It is the most common rheumatic disease in childhood, affecting approximately 1 in 1000 children [1]. The disease course can be unpredictable, and children commonly experience both physical (eg, pain, fatigue, stiffness) and emotional (eg, stress, anxiety, depression) symptoms that may restrict day-to-day function [2-7]. Persistent pain is common and can contribute to adverse psychological effects and functional disability [8,9]. As children mature, they are expected to assume increasing responsibility for disease self-management concomitant with their growing independence and autonomy. However, adherence to disease management is typically suboptimal [10,11]. Poor adherence and inappropriate strategies for self-management may reduce the potential benefits of treatment and impact health-related quality of life (HRQL) [10,11].

Improved disease self-management early in the disease trajectory may help to improve health outcomes. Web-based self-management interventions are a promising strategy for improving the accessibility and availability of education, psychosocial interventions, and social support for adolescents with JIA [12,13]. There is preliminary evidence that self-management interventions can improve symptoms and health status in certain childhood illnesses [14-20].

*Teens Taking Charge* is a Web-based treatment that was developed to meet the need for a high-quality, accessible, empirically grounded, and multilingual self-management program for adolescents with JIA [21-23]. The program was developed using a sequential phased approach. In phase 1, a qualitative needs assessment was conducted to identify the self-management needs of adolescents with JIA [21]. Adolescents articulated a universal need for more JIA-specific knowledge, self-management strategies, and meaningful social support to better manage their JIA. In phase 2, a prototype website was developed, and it underwent iterative cycles of usability testing to ensure ease of use and understanding of the program [22]. In phase 3, a pilot feasibility randomized controlled trial (RCT) was completed with 46 adolescents with JIA and their caregivers [23]. The pilot RCT supported the feasibility (high acceptability, user satisfaction, and compliance) and initial effectiveness of the program in improving knowledge and decreasing pain in adolescents with JIA compared with an education control condition.

### Objective

**Figure 1:**
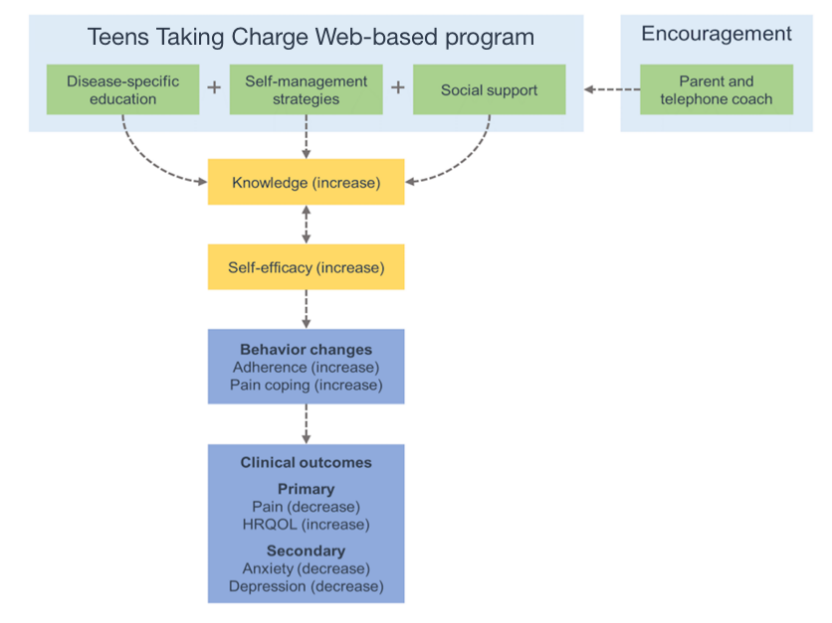
Conceptual model outlining the hypothesized mechanism of action of the Teens Taking Charge: Managing JIA Online Web-based intervention. JIA: juvenile idiopathic arthritis. HRQL: health-related quality of life.

The objective of this study was to evaluate the effectiveness of the *Teens Taking Charge* Web-based self-management intervention in reducing symptoms and improving HRQL in adolescents with JIA compared with a Web-based education control condition. We hypothesized that adolescents randomized to the intervention group would demonstrate (1) reduced pain and improved HRQL; (1) reduced emotional (anxiety and depression) symptoms; and (3) increased treatment adherence, pain coping, JIA-specific knowledge, and self-efficacy compared with adolescents randomized to the control group. See Figure 1 for a summary of the hypothesized mechanism of action of the *Teens Taking Charge* intervention on health outcomes. This model is based on the published work of Murray et al [24].

## Methods

### Trial Design

A 2-arm parallel group RCT design was conducted to evaluate the effectiveness of the *Teens Taking Charge* program in comparison with a Web-based education control condition. The allocation ratio was 1:1. The trial was registered on ClinicalTrials.gov with the identifier, NCT01572896. The study was approved by the local research ethics boards at each of the participating institutions.

### Participants

Adolescents were recruited in person from 11 Canadian pediatric rheumatology centers. A local research coordinator explained the study to eligible adolescents and obtained written consent.

Parents and caregivers were invited to participate with their child. Adolescents were eligible to participate if they were (1) between 12 and 17.9 years of age, (2) diagnosed with JIA as per their rheumatologist, (3) able to speak and read English or French, and (4) able to complete Web-based outcome measures as per self-report. Adolescents were excluded if they had (1) major comorbid illnesses or cognitive impairments that could affect their ability to use and understand the Web-based program as per their health care provider or (2) were currently participating in other cognitive behavioral therapy interventions. Parents and caregivers were eligible if they were (1) able to speak and read English or French and (2) able to complete parent modules and outcome measures. Participants received gift cards (total value Can $100 [US $75]) in recognition of their time and commitment to the study_._

### Study Conditions

#### Intervention Condition

The intervention was based on cognitive behavioral principles of improving pain and HRQL through providing disease education, empowering patients with coping skills to help manage disease symptoms and stress, and providing validation and encouragement through opportunities for social support.

The intervention condition had three components: (1) a 12-module adolescent website consisting of JIA-specific education, self-management strategies, and social support, (2) telephone health coaches for the adolescent only, and (3) a 2-module caregiver website consisting of education about the impact of JIA and strategies to support their adolescent in self-management.

**Figure 2:**
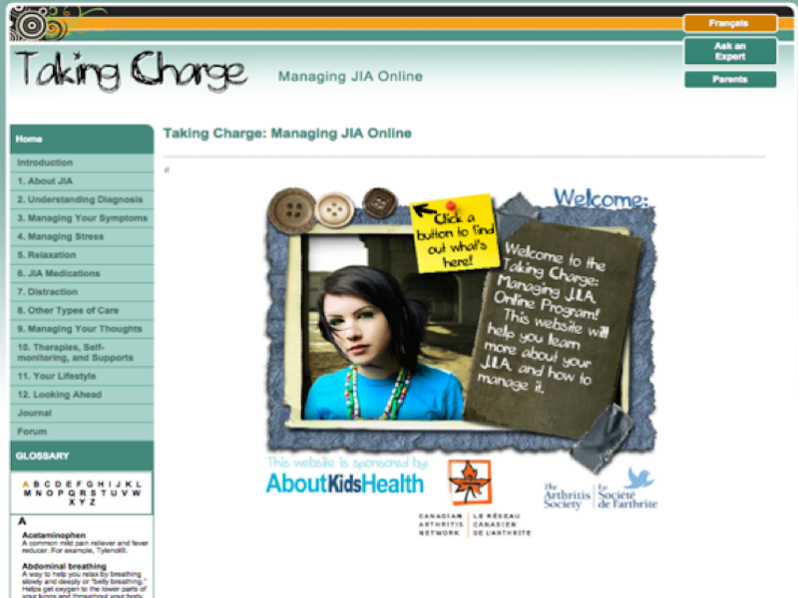
An example screenshot of the Teens Taking Charge: Managing JIA Online adolescent Web intervention.

The adolescent and caregiver websites were available in English and French. The module content is summarized in Table 1. All content was developed by a team of experts from across Canada including study investigators, allied health team members, and adolescent medicine health care professionals, and patient advocates. Modules were written at a grade 6 to grade 7 level, were developmentally appropriate for those aged 12 to 18 years, and were geared to address the needs identified in the phase 1 qualitative study [21]. See Figure 2 for screenshots of the *Teens Taking Charge* adolescent website.

Participants were instructed to work through the modules over a 12-week period at their own pace to allow sufficient flexibility to accommodate for exams, holidays, and illnesses. Participants received automated standard weekly emails to remind them to complete their next module and to congratulate them on module completion.

Adolescents in the intervention group also received monthly telephone calls from a health coach who was a trained non–health care professional [23]. The telephone coach (1) reviewed content from the previous 4 weeks (eg, assignments, knowledge quizzes, and goals); (2) determined whether the participant completed the modules and answered questions regarding the material and or self-management skills; and (3) provided guidance and helped problem-solve any issues with the program content that may have arisen in the previous 4 weeks. If participants asked questions that the coach could not answer, the coach redirected them to their rheumatology health care team. All calls were audio recorded and audited every 3 months to ensure integrity of the intervention delivery.

**Table 1:** Module breakdown of the Teens Taking Charge intervention for adolescents and caregivers.

Module number and title		Content description	
**Adolescent modules**			
	1. About JIA^a^		Clinical features of JIA
	2. Understanding diagnosis		How JIA is diagnosed, coping, and health check-ups
	3. Managing your symptoms		Understanding and managing pain, fatigue, and stiffness
	4. Managing stress		Understanding and managing stress
	5. Relaxation		Belly breathing, relaxation with and without tension, mini relaxation, and behavioral reversal
	6. JIA medications		Overview of medications for JIA and medication management
	7. Distraction		Attention focusing, imagery, mental games, and pleasant activities
	8. Other types of care		Physical activity, nutrition, orthotics, occupational therapy, psychological therapy, complementary therapy, surgical procedures, eye care, and dental care
	9. Managing your thoughts		Understanding stress and thinking; changing negative thoughts
	10. Therapies, self-monitoring, and supports		Getting the most from your treatment, self-monitoring, and communication with the health care team
	11. Your lifestyle		Staying active, healthy eating, and lifestyle choices
	12. Looking ahead		Transitioning to adult care, preparing for higher education and employment, and maintaining a treatment program
**Caregiver modules**			
	1. Impact of arthritis		Impact of JIA on the family and finances; JIA disease education
	2. Letting go		Communicating with your teen and helping your teen to take control of disease management

^a^JIA: juvenile idiopathic arthritis.

#### Education Control Condition

This condition was designed to control for the potential effects on outcomes of time, attention, and computer use during the intervention and through the follow-up period. In addition to standard medical care, adolescents and caregivers in the education control group were provided with access to a self-guided education study website called the *JIA Resource Centre*. It featured links to 12 publicly available JIA websites (identified from a systematic review) that provided patient education, but did not offer self-management strategies or opportunities for social support [25]. The information in these websites was similar to the materials that are routinely provided at the time of diagnosis in the participating recruitment centers. The educational websites were monitored on a weekly basis to ensure that they did not add any *active ingredients* during the trial. See Multimedia Appendix 1 for a list of websites in the *JIA Resource Centre.* Adolescents and caregivers were encouraged to browse one website per week from the *JIA Resource Centre*. They received automated email reminders each week to access the education content.

Each adolescent in the education control group also received monthly telephone calls from a coach. The coaches were trained non–health care professionals who encouraged participants to view the websites and also discussed adolescents’ *own best efforts* at managing their JIA over the 12-week period. The coach used standardized scripts for these calls. They did not provide any advice or self-management support to participants, and the interaction was minimal. If a participant asked for information related to self-management of their JIA, the coach redirected them to their rheumatology health care team. All calls were recorded and audited to ensure integrity of the control calls. Participants from the education control group were offered the full intervention following their trial completion (at 12-month time point after completion of all outcome measures) for a period of 3 months.

#### Outcomes

Outcome data included a combination of adolescent self-reports and parent-proxy reports, as outlined in Multimedia Appendix 2 [26-34]. Measures were completed at four different time points: baseline (after consent, before randomization; T_1_), condition completion (3 months after randomization; T_2_), 6 months after randomization (T_3_), and 12 months after randomization (T_4_). Measures were completed online by study participants through the Research Electronic Data Capture secure Web-based system hosted at the Hospital for Sick Children. The lead study coordinator contacted participants via telephone, email, and mail to remind them to complete follow-up measures. All measures have evidence of validity and reliability for adolescents with arthritis and were available in English and French.

#### Sample Size

Sample size was calculated by assuming an effect size (standardized group difference) of 0.31 units on the outcome of average pain intensity over a 12-month follow-up because this magnitude of difference is considered the minimally clinically significant important group difference for pain intensity [35]. It is also congruent with the treatment effect observed during pilot work [23]. Assuming a type I error rate (alpha) of .05 and the maximum correlation between repeated pain measurements to be 0.90, a sample size of 117 participants per group (234 in total) was required to achieve 80% power to detect an effect of this magnitude or larger. To account for the lack of compliance in completing online measures, we assumed a loss to follow-up of 20% based on our pilot RCT (13%) [23] and systematic review [36]. Therefore, we planned to randomize 294 adolescents to one of the two groups.

#### Randomization

Randomization was centrally controlled, concealed, and balanced by physician-rated disease activity into those with low (<3/10) and moderate-to-severe (>4/10) disease activity [37] and by study center (ie, to control for center-specific education, transitional care programs, and treatment approaches). A secure, Web-based randomization service was used for allocating participants to the trial groups. Following randomization, participants were sent instructions on how to access their assigned program (website link and log-in) and were contacted by their assigned coach.

#### Blinding

This was a single blind study (adolescent and caregiver only). Both groups received a website and monthly telephone calls. Participants were informed that they would be chosen at random to either receive the *Taking Charge: Managing JIA Online* internet education program or the *JIA Resource Centre* internet education program. They were not explicitly told which program was considered the intervention condition.

#### Statistical Methods

As per an intent-to-treat approach, all participants were included in the final analysis and according to the arm (intervention or control) to which they were randomized. Only participants who completed at least one follow-up outcome assessment after baseline were included in the analysis. In addition, outcome assessments were required to be completed within 35 days of each time point (ie, 3 months, 6 months, 12 months) to be included in the analysis for that time point. This 35-day window was used because the *PedsQL Rheumatology Module* asks respondents to describe their function over the previous month [38]. Thus, participants who completed their outcome assessments beyond the assigned time point were reporting their function within a different window of time compared with participants who completed assessments on schedule and the data were considered missing.

Data were analyzed using the SAS software (Version 9.1.3. developed by SAS Institute Inc, Cary, North Carolina, 2006). [39]. Descriptive statistics were used to describe characteristics of the sample. A significance level of .05 was used for the primary question. A Bonferroni-adjusted alpha level of .007 was used to maintain an overall level of .05 for all secondary and other study outcomes. Linear mixed models were used to assess the effects of the intervention on primary, secondary, and other outcomes using the baseline scores as covariates. Models used an autoregressive first order covariance structure, which allows for correlations between measurements to decline as they are further apart in time [40,41]. To assess the effects of disease-related variables (eg, disease activity, duration of illness, and disease subtype) and age on primary and secondary outcomes, separate linear mixed models of each posttreatment measure were constructed using pretreatment disease-related variables and age as covariates [42].

#### Data Safety and Monitoring

A Data Safety and Monitoring Board (DSMB) was formed to regularly review the safety and treatment-specific efficacy data of the trial. The board included experts in translational research, pediatric rheumatology, chronic pain, cognitive behavioral therapy, RCTs, and biostatistics. The DSMB met via teleconference every 6 months for the duration of the trial.

## Results

### Participants

**Figure 3:**
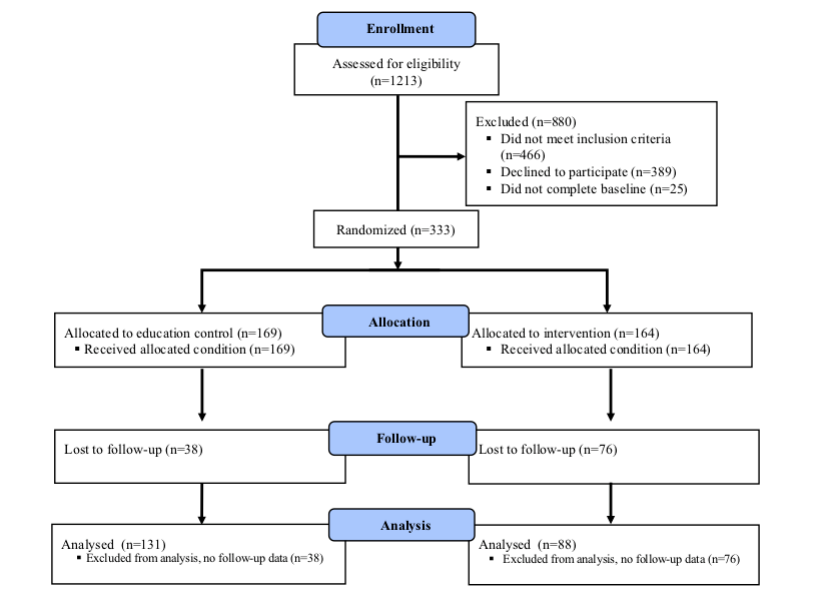
Consolidated Standards of Reporting Trials flow diagram.

The Consolidated Standards of Reporting Trials (CONSORT) [43] flow diagram detailing participant enrolment, allocation, follow-up, and analysis is provided in Figure 3. The randomized study sample consisted of 333 adolescents. As illustrated in the CONSORT diagram, 76/164 (46.3%) participants in the intervention group and 38/169 (22.5%) in the control group were lost to follow-up. Those participants lost to follow-up did not have any significant differences in baseline characteristics with the exception of the PedsQL worry subscale (lost to follow-up=40.0; analytic=45.9; *P*=.03).

Across the entire sample, 100/604 (16.6%) outcome assessments were completed beyond the 35-day window for that time point. The breakdown of overdue assessments across time points was as follows: 3 months (24/197, 12.2%); 6 months (38/200, 19.0%); and 12 months (38/207, 18.4%). Comparing the groups, 45/251 (17.9%) intervention outcome assessments and 55/353 (15.6%) control outcome assessments were completed outside the window. Similarly, 101/541 (18.7%) caregiver outcome assessments were completed beyond the 35-day window for that time point. The breakdown of overdue caregiver assessments across time points was: 3 months (26/169, 15.4%); 6 months (35/182, 19.2%); 12 months (40/190, 21.1%). Comparing the caregiver study groups, 46/216 (21.3%) intervention assessments and 55/325 (16.9%) control assessments missed the allowed window.

Characteristics of the analyzed adolescent sample by assigned condition are provided in Table 2. The analyzed caregiver sample consisted of biological mothers (158/197, 80.2%), biological fathers (36/197, 18.3%), and adoptive parents or grandparents (3/197, 1.5%). More than half of the caregiver sample (129/197, 65.5%) had graduated from college or graduate school.

**Table 2:** Adolescent demographic and disease characteristics.

Characteristic		Total (N=219)		Intervention (n=88)		Control (n=131)	
Age (years), mean (SD)		14.4 (1.6)		14 (1.5)		14.5 (1.7)	
**Sex, n (%)**							
	Female		154 (70.3)		63 (72)		91 (69.5)
	Male		65 (29.7)		25 (28)		40 (30.5)
**Language, n (%)**							
	English		214 (97.7)		87 (99)		127 (96.9)
	French		5 (2.3)		1 (1)		4 (3.1)
**Annual household income (Can $), n (%)^a^**							
	Less than $25,000 (US $18,263)		9 (4.6)		2 (3)		7 (5.7)
	$25,000-49,999 (US $18,263-US $36,526)		25 (12.7)		9 (12)		16 (13.1)
	$50,000-74,999 (US $36,526-US $54,790)		28 (14.2)		6 (8)		22 (18.0)
	$75,000-99,999 (US $54,790-US $73,053)		29 (14.7)		13 (17)		16 (13.1)
	$100,000-150,000 (US $73,053-US $109,580)		33 (16.8)		12 (16)		21 (17.2)
	Did not answer		44 (22.3)		18 (24)		26 (21.3)
**JIA^b^ category, n (%)**							
	Systemic		5 (2.3)		1 (1)		4 (3.1)
	Oligoarthritis		47 (21.5)		21 (24)		26 (19.8)
	Oligoarthritis—extended		24 (10.9)		9 (10)		15 (11.5)
	Polyarthritis (RF-)		50 (22.8)		23 (26)		27 (20.6)
	Polyarthritis (RF+)		19 (8.7)		5 (6)		14 (10.7)
	Psoriatic arthritis		23 (10.5)		9 (10)		14 (10.7)
	Enthesitis-related arthritis		35 (16.0)		13 (15)		22 (16.7)
	Undifferentiated		9 (4.1)		3 (3)		6 (4.6)
	Other		7 (3.2)		4 (5)		3 (2.3)
**Disease severity, n (%)**							
	Low (0-3 PGA)^c^		181 (82.6)		75 (85)		106 (80.9)
	Moderate to severe (4-10 PGA)		38 (17.4)		13 (15)		25 (19)
Duration of illness (years), mean (SD)		5.7 (4.6)		6 (5)		5.6 (4.6)	
Expectation about intervention effectiveness at baseline		6.1 (2.1)		6 (2)		5.9 (2.0)	

^a^Parent report, N=197.

^b^JIA: juvenile idiopathic arthritis.

^c^PGA: physician global assessment [23].

### Adherence to Assigned Study Condition

Telephone coach calls were considered *complete* if a participant had reviewed their assigned website module content for that month. Thus, coach calls were used to define participant adherence to their assigned condition. In total, 72.4% (241/333) of participants completed at least two of three coach calls. Across all participants, coach call completion was as follows: 0 calls (75/333, 22.5%); 1 call (17/333, 5.1%); 2 calls (4/333, 1.2%); or 3 calls (237/333, 71.2%). A higher proportion of participants in the control condition met the minimum criteria for being considered adherent compared with the intervention condition (148/169, 87.6% vs 93/164, 56.7%). Overall, 146/169 (86.4%) control participants and 91/164 (55.5%) intervention participants completed all 3 calls.

### Primary and Secondary Outcomes: Adolescents

**Figure 4:**
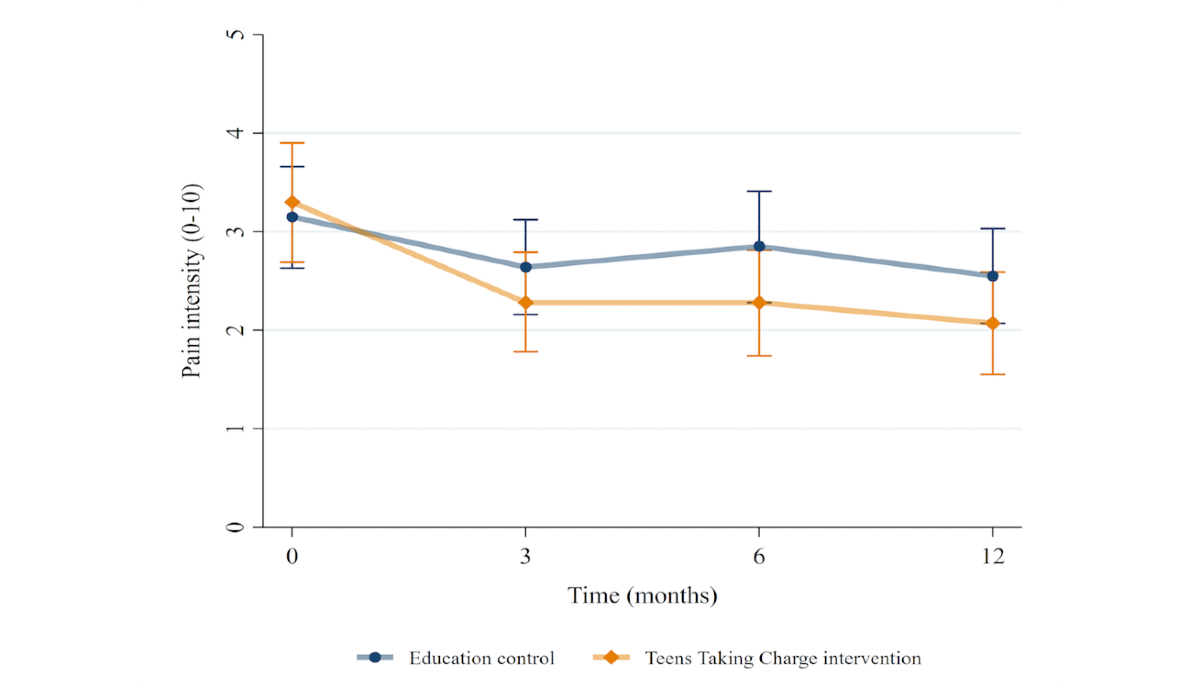
Pain intensity scores over time in each treatment group, mean and 95% CI.

**Figure 5:**
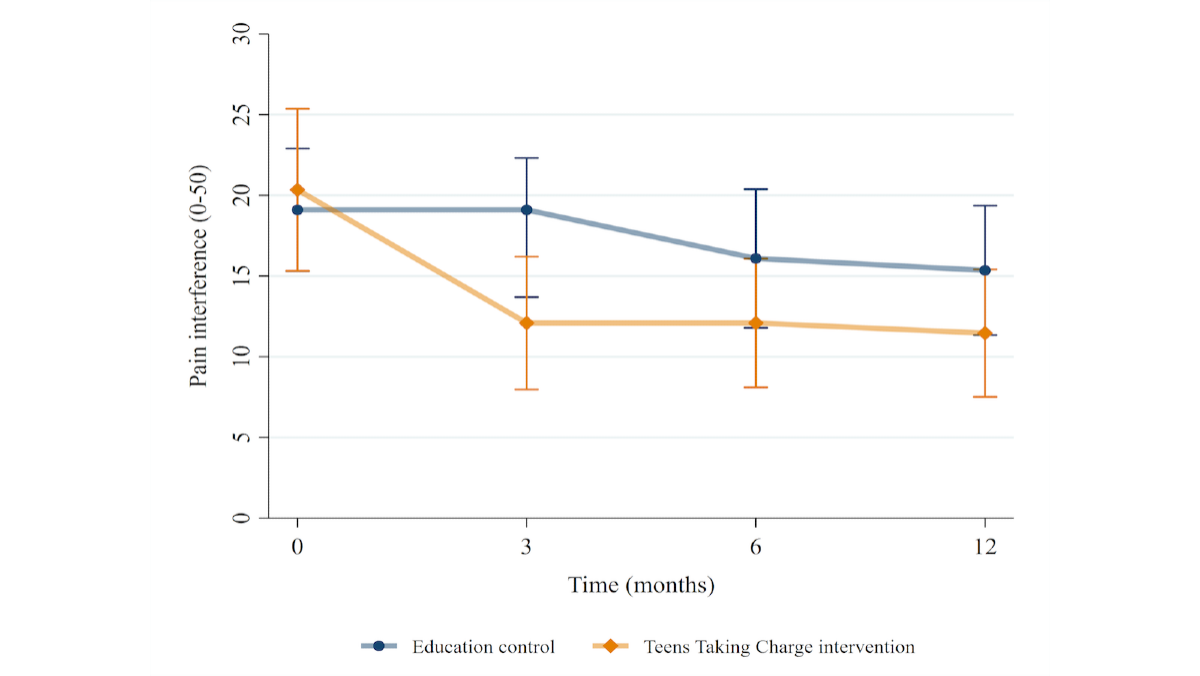
Pain interference scores over time in each treatment group, mean and 95% CI.

**Figure 6:**
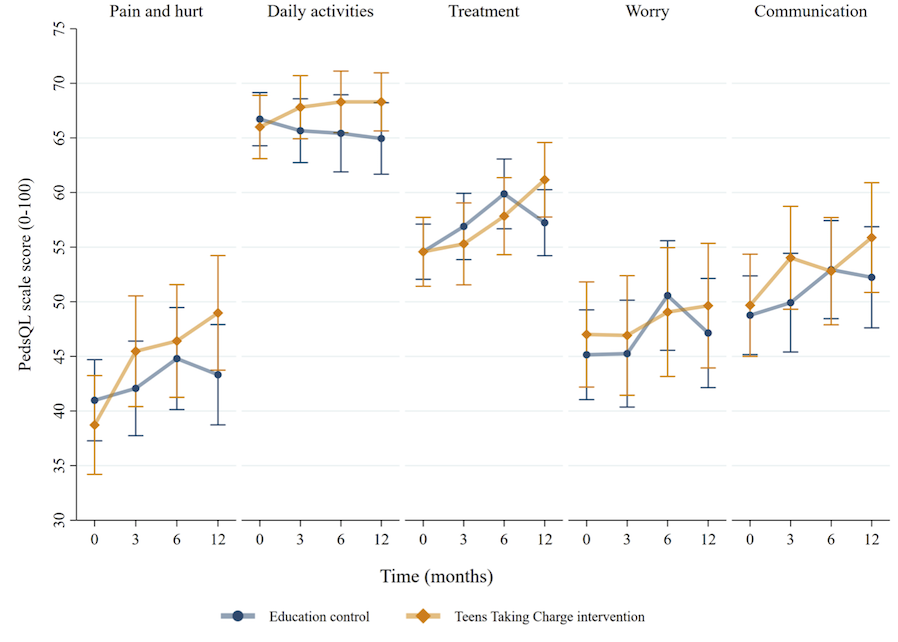
PedsQL subscale scores over time in each treatment group, mean and 95% CI.

There was a significant effect of condition on pain intensity (*P=*.02) and pain interference (*P=*.007) after adjusting for baseline differences between the groups, with improved scores (lower values) observed in the *Teens Taking Charge* group. The effect of condition was stable over time (from 3 to 12 months), as there were no significant conditions by time interactions. Similar results were seen for the HRQL domains of problems with pain (*P=*.02) and problems with daily activities (*P=*.01). Although there was no overall condition effect, there was a significant condition by time interaction (*P=*.008) for treatment problems, suggesting that the difference between the groups changed over time. No significant differences were seen on the PedsQL subscales of worry or communication problems. Results from the linear mixed models are presented in Table 3, and the mean values in both groups over time are depicted in Figures 4 to 6. See Multimedia Appendices 3-5 for additional analytic outputs from the adolescent analyses.

**Table 3:** Adolescent reported primary outcomes, linear mixed models.

Outcome measure			Linear mixed model										
			Time				Condition				Time by condition		
			*F* value (*ddf*^a^=281)		*P* value		*F* value (*ddf*=281)		*P* value		*F* value (*ddf*=281)		*P* value
**Pain**													
	Pain intensity	1.12		.33		5.44		.02		0.49		.61	
	Pain interference	1.23		.30		7.40		.007		0.40		.67	
**Quality of life**													
	Problems with pain	3.68		.03		5.40		.02		0.50		.61	
	Problems with daily activities	0.12		.89		6.39		.01		0.19		.83	
	Treatment problems	4.30		.02		0.12		.73		4.94		.008	
	Worry	2.64		.07		0.43		.51		0.20		.82	
	Communication problems	2.93		.06		1.49		.22		0.42		.66	

^a^ddf: denominator degrees of freedom.

### Primary and Secondary Outcomes: Caregivers

The primary and secondary caregiver outcome variables as a function of condition and assessment time point are displayed in Multimedia Appendices 6 and 7. There were no significant differences between study groups on primary and secondary caregiver outcomes.

## Discussion

### Principal Findings

This study sought to evaluate the effectiveness of the *Teens Taking Charge* intervention compared with Web-based education in improving symptoms and HRQL in adolescents with JIA. Our results indicate that the *Teens Taking Charge* self-management intervention has evidence of effectiveness at improving pain intensity and pain interference. These improvements were sustained for up to 12 months postrandomization. The intervention group also demonstrated significant improvements in HRQL related to pain and hurt, daily activities, and treatment problems compared with the control. There were improvements, yet no significant group differences in the secondary outcomes of emotional symptoms, treatment adherence, pain coping, JIA knowledge, and self-efficacy. The data trends support the conceptual model for how JIA self-management education could be foundational to promote improvement in health outcomes.

### Comparison With Previous Work

As expected, there is concordance between the results of this trial and the *Teens Taking Charge* pilot RCT. The pilot trial demonstrated initial program effectiveness in improving JIA knowledge and decreasing pain intensity compared with education control [23].

Results of this RCT should also be compared with published results by Connelly et al [44], who evaluated an adapted version of *Teens Taking Charge* in English and Spanish-speaking adolescents with JIA in the United States. Data from the Connelly trial indicated that participants in both study groups had comparable and statistically significant improvements in pain intensity, pain interference, and HRQL over the study period, with no significant between-group differences. The effect size estimates associated with outcome improvement were small.

The findings of these two RCTs should be considered in the context of several factors. First, this study was conducted in Canada while the Connelly study was conducted in the United States. Given that the *Teens Taking Charge* program was delivered in addition to usual care, it is possible that regional differences in baseline care affected the relative impact of the supplemental intervention. Although both RCTs demonstrated improvements in primary outcomes in the control and intervention groups, only this study found significant improvements in the intervention participants compared with control participants. Connelly hypothesized that the choice to compare a self-directed Web-based self-management intervention with an *active* control arm (ie, online education) rather than a wait list or standard care condition may have contributed to the lack of between-group differences in his trial. Participants may have engaged differently with the *active control* condition—with participants in the American trial benefiting more from the provided JIA education and/or attention from a telephone coach. This may be a function of the type of education routinely offered as part of usual clinic care and/or how much participants actually viewed and used the *active control* content. Unfortunately, as data about how often control participants perused the educational websites were not captured, direct comparisons between the trials in this regard is not possible.

This trial’s results also concur with two systematic reviews, which found that remotely delivered self-management interventions can be effective in improving painful symptoms in children and adolescents [45,46]. However, a meta-analysis of pediatric chronic pain studies [46], which used an active comparator condition rather than usual care, found that Web-based cognitive behavioral therapy was not significantly better than active control (pooled *g*=0.10; 95% CI −0.32 to 0.52; *P*=.64). Although this study did demonstrate significant between-group differences with an active comparator group, our findings should be considered in the context of previous similar studies. Of the three active control studies included in the meta-analysis study [46], the trial by Palermo et al [47] had the largest sample size (N=273 vs N=18 and N=48) and the highest Moncrieff quality rating [48] (37 vs 16 and 26, respectively). The Palermo trial evaluated a program called Web-based Management of Adolescent Pain (Web-MAP2). Their data demonstrated that, from baseline to 6-month follow-up, adolescents with chronic pain who received the Web-MAP2 intervention achieved greater reductions in the primary outcome of daily activity limitations than the education control group. However, while both groups improved, there was no significant difference between groups from baseline to the immediate posttreatment period. Most participants in the Palermo trial reported a baseline pain intensity of moderate-to-severe intensity, while most participants in this trial (181/219, 82.6%) had low disease severity at baseline and low pain intensity (see Table 2). As they started their trial with higher dysfunction, intervention participants in the Palermo trial may have needed more time to consolidate and apply their learned self-management skills, which may have contributed to a lack of differentiation in initial pain outcomes compared with control group participants who received pain education alone.

Overall, this study demonstrates that a Web-delivered cognitive behavioral intervention can significantly improve health outcomes in adolescents with JIA, compared with active control, and that these improvements can be sustained from the immediate posttreatment period for up to 12 months.

### Trial Strengths

The extended follow-up period over 1 year allowed for the examination of the maintenance of treatment effects. This is an important aspect of trial quality to address in pediatric Web-based self-management interventions, given that most previous studies have only reported outcomes in the immediate posttreatment period. The use of an education control condition is also an important contribution as most pediatric Web-based RCTs have employed usual care or wait-list control conditions, making it difficult to separate the effects of active treatment from those of increased attention and access to an online program.

### Trial Limitation

In this study, telephone coach calls were used as a proxy for program adherence. The website platform, which was built and supported by hospital IT infrastructure, did not support the capture of user-level usage analytics at the time of development. Thus, the impact of program usage level on outcomes could not be examined.

As per the sample size calculation, this trial aimed to analyze a minimum of 117 participants per group. The study analytic sample included 88 intervention and 131 control participants. This loss of participants reduced our statistical power to detect small differences between the study groups. Although we did identify significant group differences on primary outcomes owing to sufficiently large effect sizes, it is possible that smaller effect sizes on other outcomes were missed owing to the underpowered sample.

The higher rate of loss to follow-up in the intervention vs control group may have been the result of increased participant burden (eg, greater time commitment required to review intervention content vs control). Intervention group participants were also less likely to complete all coach telephone coach calls than those in the control group. In light of the high rate of loss to follow-up and lack of data on program usage, the generalizability of study findings should be interpreted with caution.

### Significance

This study shows the beneficial effects of a digital approach to disease self-management for the youth with JIA. This paradigm could have far-reaching implications for self-management of other chronic health conditions experienced by the youth. Given that the *Teens Taking Charge* program has been found effective, we envision that it should be offered as a resource for all youths with JIA to supplement their medical care. We believe that this adjunctive self-guided online program will help to overcome current barriers that prevent adolescents with JIA and their families from receiving adequate support in disease self-management.

### Conclusions and Next Steps

The results of this RCT indicate that the *Teens Taking Charge* Web-based intervention is effective at reducing both pain intensity and pain interference and improving HRQL in adolescents with JIA compared with education control. These effects are sustained for up to 12 months following program completion. The *Teens Taking Charge* program is now publicly available at no cost [49]. Efforts are underway to widely disseminate this resource to increase the accessibility of self-management care for adolescents and families living with JIA. Efforts are also underway to culturally adapt the intervention for use in different settings.

## Appendix

Multimedia Appendix 1 Publicly accessible arthritis education websites provided in the “JIA Resource Centre" for education control participants.

Multimedia Appendix 2 Measures.

Multimedia Appendix 3 Adolescent reported primary outcomes over time by condition.

Multimedia Appendix 4 Adolescent reported secondary outcomes over time by condition.

Multimedia Appendix 5 Adolescent reported secondary outcomes, linear mixed models.

Multimedia Appendix 6 Parent-reported outcomes over time by condition.

Multimedia Appendix 7 Parent reported outcomes, linear mixed models.

Multimedia Appendix 8 CONSORT-eHEALTH checklist (V 1.6.1).

## Abbreviations

CONSORT: Consolidated Standards of Reporting Trials
DSMB: Data Safety and Monitoring Board
HRQL: health-related quality of life
JIA: juvenile idiopathic arthritis
RCT: randomized controlled trial
Web-MAP2: Web-based Management of Adolescent Pain
