# Supplementary material for: Teens Taking Charge: A Randomized Controlled Trial of a Web-Based Self-Management Program With Telephone Support for Adolescents With Juvenile Idiopathic Arthritis
Source: J Med Internet Res. 2020 Jul 29;22(7):e16234. doi: 10.2196/16234 (PMC7424488; doi:10.2196/16234)
Supplement: Multimedia Appendix 1 [file jmir_v22i7e16234_app1.docx]

| **Module** | **Link** |
| --- | --- |
| 1 | WebMD (English) or Pediatric Rheumatology International Trials Organization (French) |
| 2 | MayoClinic |
| 3 | eMedicineHealth |
| 4 | Keep Kids Healthy |
| 5 | Children’s Chronic Arthritis Association (CCAA) Kids with Arthritis |
| 6 | Arthritis Research Campaign |
| 7 | Orthopaedic Surgery and Sports Medicine at the University of Washington |
| 8 | The Arthritis Society, Just for Kids |
| 9 | Wikipedia Encyclopedia |
| 10 | Virtual Medicine Centre |
| 11 | American College of Rheumatology |
| 12 | Cedars-Sinai Health System |
