# Supplementary material for: Teens Taking Charge: A Randomized Controlled Trial of a Web-Based Self-Management Program With Telephone Support for Adolescents With Juvenile Idiopathic Arthritis
Source: J Med Internet Res. 2020 Jul 29;22(7):e16234. doi: 10.2196/16234 (PMC7424488; doi:10.2196/16234)
Supplement: Multimedia Appendix 2 [file jmir_v22i7e16234_app2.docx]

| Outcome | | Measure | Description | Respondent | Time point administered | | | |
| --- | --- | --- | --- | --- | --- | --- | --- | --- |
|  |  |  |  |  | T1 | T2 | T3 | T4 |
|  | | | | | | | | |
| **Background** | | | | | | | | |
|  | Participant characteristics | Background questionnaire (investigator developed) | Sociodemographic variables, JIA characteristics^a^, and duration | Adolescent and parent | X | — | — | — |
|  | Treatment expectancy | Treatment Expectancies Scale [26] | Assesses expectations of program helpfulness using an 11-point NRS^b^ with anchors: 0=“don’t think it will help at all”; 10=“think it will help a lot” | Adolescent and parent | X | — | — | — |
|  | Disease activity | Physician Global Assessment [27] | Assesses current disease activity on a 0- to 10-point NRS with anchors of “no activity” and “maximum activity” | Physician | X | — | — | — |
| **Primary outcomes** | | | | | | | | |
|  | Pain | Recalled Pain Inventory—Short Form [28] | Assesses average pain intensity over the past week on a 0- to 10-point NRS with higher scores indicating worse pain | Adolescent | X | X | X | *X* |
|  | HRQL | PedsQL Rheumatology Modules [29] | Assesses pain and hurt, daily activities, treatment, worry, and communication over the past month. Items are rated on a 5-point Likert scale ranging from “never” to “almost always” | Adolescent and parent | X | X | X | *X* |
| **Secondary outcomes** | | | | | | | | |
|  | Anxiety | PROMIS^c^ Pediatric Anxiety Short-Form [30] | Assesses emotional functioning related to anxiety over past 7 days. Items are rated on a 5-point Likert scale ranging from “never” to “almost always” | Adolescent | X | X | X | *X* |
|  | Depression | PROMIS Depressive Symptoms Short Form [30] | Assesses emotional functioning related to depression over past 7 days. Items are rated on a 5-point Likert scale ranging from “never” to “almost always” | Adolescent | X | X | X | *X* |
|  | Adherence to medical treatment | Child Adherence Report Questionnaire and Parent Adherence Report Questionnaire [31] | Assesses adherence to prescribed medications, exercises, and wearing of splints over the past 3 months | Adolescent and parent | X | X | X | *X* |
|  | Pain coping | Pain Coping Questionnaire [32,33] | Assesses how often a given type of coping strategy is used when respondent is in pain for a few hours or days. Items are rated on a 5-point Likert scale ranging from “never” to “very often” | Adolescent | X | X | X | *X* |
|  | JIA knowledge | Medical Issues, Exercise, Pain and Social Support Questionnaire [34] | Knowledge items are rated on an 11-point NRS ranging from “none at all” to “enough” | Adolescent and parent | X | X | X | *X* |
|  | Self-efficacy | Children’s Arthritis Self-Efficacy; Parent’s Arthritis Self-Efficacy [35,36] | Assesses certainty of ability to manage symptoms, emotional consequences and activities related to their arthritis/their child’s arthritis. Higher scores indicate greater certainty | Adolescent and Parent | X | X | X | *X* |

^a^Information on the juvenile idiopathic arthritis (JIA) diagnosis was captured from the medical chart.

^b^NRS: numerical rating scale.

^c^PROMIS: Patient Reported Outcomes Measurement Information System.

**References**

26. Goossens MEJB, Vlaeyen JWS, Hidding A, Kole-Snijders A, Evers SMAA. Treatment expectancy affects the outcome of cognitive-behavioral interventions in chronic pain. Clin J Pain. 2005 Feb;21(1):18–26; discussion 69-72.

27. Moretti C, Viola S, Pistorio A, Magni-Manzoni S, Ruperto N, Martini A, Ravelli A. Relative responsiveness of condition specific and generic health status measures in juvenile idiopathic arthritis. Ann Rheum Dis 2005 Feb;64(2):257–261. PMID:15647433

28. Stinson JN, Stevens BJ, Feldman BM, Streiner D, McGrath PJ, Dupuis A, Gill N, Petroz GC. Construct validity of a multidimensional electronic pain diary for adolescents with arthritis. Pain 2008 Jun;136(3):281–292. PMID:17723279

29. Varni JW, Seid M, Smith Knight T, Burwinkle T, Brown J, Szer IS. The PedsQL in pediatric rheumatology: reliability, validity, and responsiveness of the Pediatric Quality of Life Inventory Generic Core Scales and Rheumatology Module. Arthritis Rheum. 2002 Mar;46(3):714–25.

30. Irwin DE, Stucky B, Langer MM, Thissen D, Dewitt EM, Lai J-S, Varni JW, Yeatts K, DeWalt DA. An item response analysis of the pediatric PROMIS anxiety and depressive symptoms scales. Qual Life Res 2010 May;19(4):595–607. PMID:20213516

31. Feldman DE, de Civita M, Dobkin PL, Malleson P, Meshefedjian G, Duffy CM. Perceived adherence to prescribed treatment in juvenile idiopathic arthritis over a one-year period. Arthritis Rheum. 2007 Mar 15;57(2):226–33.

32. Reid GJ, Gilbert CA, McGrath PJ. The Pain Coping Questionnaire: preliminary validation. Pain. 1998 May;76(1–2):83–96.

33. Huguet A, Miró J, Nieto R. The factor structure and factorial invariance of the Pain-Coping Questionnaire across age: evidence from community-based samples of children and adults. Eur J Pain Lond Engl. 2009 Sep;13(8):879–89.

34. André M, Hedengren E, Hagelberg S, Stenström CH. Perceived ability to manage juvenile chronic arthritis among adolescents and parents: development of a questionnaire to assess medical issues, exercise, pain, and social support. Arthritis Care Res Off J Arthritis Health Prof Assoc. 1999 Aug;12(4):229–37.

35. Barlow JH, Shaw KL, Wright CC. Development and preliminary validation of a children’s arthritis self-efficacy scale. Arthritis Rheum. 2001 Apr;45(2):159–66.

36. Barlow JH, Shaw KL, Wright CC. Development and preliminary validation of a self-efficacy measure for use among parents of children with juvenile idiopathic arthritis. Arthritis Care Res Off J Arthritis Health Prof Assoc. 2000 Aug;13(4):227–36.
