# Supplementary material for: Teens Taking Charge: A Randomized Controlled Trial of a Web-Based Self-Management Program With Telephone Support for Adolescents With Juvenile Idiopathic Arthritis
Source: J Med Internet Res. 2020 Jul 29;22(7):e16234. doi: 10.2196/16234 (PMC7424488; doi:10.2196/16234)
Supplement: Multimedia Appendix 3 [file jmir_v22i7e16234_app3.docx]

| **Outcome** | **Teens Taking Charge intervention** | | | | | | | | **Education control** | | | | | | | |
| --- | --- | --- | --- | --- | --- | --- | --- | --- | --- | --- | --- | --- | --- | --- | --- | --- |
|  | **Baseline** | | **3 month** | | **6 month** | | **12 month** | | **Baseline** | | **3 month** | | **6 month** | | **12 month** | |
|  | **N** | **Mean (SD)** | **N** | **Mean (SD)** | **N** | **Mean (SD)** | **N** | **Mean (SD)** | **N** | **Mean (SD)** | **N** | **Mean (SD)** | **N** | **Mean (SD)** | **N** | **Mean (SD)** |
| Pain |  |  |  |  |  |  |  |  |  |  |  |  |  |  |  |  |
| Intensity | 88 | 3.30 (2.86) | 67 | 2.28 (2.06) | 69 | 2.28 (2.24) | 70 | 2.07  (2.18) | 131 | 3.15 (3.00) | 106 | 2.64 (2.50) | 93 | 2.85  (2.74) | 99 | 2.55 (2.44) |
| Interference | 88 | 20.34 (23.69) | 67 | 12.09 (16.87) | 69 | 12.09  (16.60) | 70 | 11.46 (16.57) | 131 | 19.10 (21.98) | 106 | 18.00  (22.39) | 93 | 16.09  (20.86) | 99 | 15.35  (20.10) |
| Quality of life |  |  |  |  |  |  |  |  |  |  |  |  |  |  |  |  |
| Problems with pain | 88 | 38.72 (21.32 | 67 | 45.47 (20.78) | 69 | 46.41 (21.49) | 70 | 48.98  (21.99) | 131 | 40.98 (21.50) | 106 | 42.07 (22.37) | 93 | 44.80 (22.69) | 99 | 43.32 (23.03) |
| Problems with daily activities | 88 | 66.00  (13.70) | 67 | 67.81 (11.85) | 69 | 68.30  (11.74) | 70 | 68.30  (11.17) | 131 | 66.72 (14.09) | 106 | 65.66 (15.10) | 93 | 65.42 (17.14) | 99 | 64.95  (16.40) |
| Treatment problems | 88 | 54.58  (14.89) | 67 | 55.30  (15.38) | 69 | 57.84  (14.67) | 70 | 61.17  (14.30) | 131 | 54.59  (14.62) | 106 | 56.90  (15.68) | 93 | 59.88  (15.51) | 99 | 57.24  (15.14) |
| Worry | 88 | 47.01  (22.72) | 67 | 46.92  (22.45) | 69 | 49.06  (24.55) | 70 | 49.64  (23.92) | 131 | 45.15  (23.78) | 106 | 45.25  (25.28) | 93 | 50.57  (24.35) | 99 | 47.14  (25.06) |
| Communication problems | 88 | 49.68 (22.10) | 67 | 54.03 (19.31) | 69 | 52.80 (20.43) | 70 | 55.88  (21.04) | 131 | 48.77 (20.86) | 106 | 49.92  (23.34) | 93 | 52.94  (21.80) | 99 | 52.24  (23.20) |
