# Supplementary material for: Teens Taking Charge: A Randomized Controlled Trial of a Web-Based Self-Management Program With Telephone Support for Adolescents With Juvenile Idiopathic Arthritis
Source: J Med Internet Res. 2020 Jul 29;22(7):e16234. doi: 10.2196/16234 (PMC7424488; doi:10.2196/16234)
Supplement: Multimedia Appendix 4 [file jmir_v22i7e16234_app4.docx]

| **Outcome** | **Teens Taking Charge intervention** | | | | | | | | **Education control** | | | | | | | |
| --- | --- | --- | --- | --- | --- | --- | --- | --- | --- | --- | --- | --- | --- | --- | --- | --- |
|  | **Baseline** | | **3 month** | | **6 month** | | **12 month** | | **Baseline** | | **3 month** | | **6 month** | | **12 month** | |
|  | **N** | **Mean (SD)** | **N** | **Mean (SD)** | **N** | **Mean (SD)** | **N** | **Mean (SD)** | **N** | **Mean (SD)** | **N** | **Mean (SD)** | **N** | **Mean (SD)** | **N** | **Mean (SD)** |
| Anxiety | 86 | 56.52 (7.78) | 67 | 56.78 (8.16) | 68 | 57.22 (8.39) | 69 | 55.84 (8.20) | 128 | 57.19 (7.64) | 99 | 57.60 (8.79) | 91 | 55.51 (7.34) | 97 | 58.02 (9.15) |
| Depression | 87 | 58.39 (7.12) | 65 | 57.82 (6.98) | 68 | 57.79 (6.32) | 69 | 57.32 (6.28) | 123 | 57.36 (5.73) | 99 | 58.36 (7.18) | 91 | 57.66 (6.97) | 97 | 59.13 (8.44) |
| Adherence Report Questionnaire |  |  |  |  |  |  |  |  |  |  |  |  |  |  |  |  |
| Medications | 77 | 7.81 (2.28) | 52 | 8.22 (2.37) | 58 | 7.63 (3.16) | 53 | 8.40 (2.19) | 116 | 7.98 (2.53) | 84 | 7.94 (2.42) | 72 | 7.97 (2.51) | 78 | 7.94 (2.72) |
| Exercises | 60 | 6.28 (2.77) | 42 | 5.50 (2.90) | 37 | 6.50 (2.59) | 31 | 5.66 (3.07) | 85 | 6.21 (2.86) | 55 | 5.58 (2.94) | 46 | 5.99 (3.27) | 49 | 5.63 (3.19) |
| Splints | 17 | 7.06 (3.32) | 7 | 6.29 (2.75) | 8 | 7.81 (2.45) | 9 | 3.94 (2.44) | 18 | 5.14 (3.68) | 10 | 6.10 (3.11) | 9 | 7.50 (3.64) | 13 | 7.19 (3.65) |
| Pain Coping Questionnaire | 88 | 41.26 (14.26) | 66 | 42.89 (16.71) | 69 | 42.77 (18.46) | 70 | 38.67 (16.77) | 131 | 39.33 (15.67) | 104 | 39.48 (16.57) | 91 | 35.38 (18.46) | 99 | 39.74 (17.01) |
| Medical Issues Questionnaire | 88 | 46.16 (23.37) | 67 | 68.92 (20.23) | 69 | 62.25 (22.03) | 70 | 65.29 (23.62) | 131 | 47.88 (23.25) | 104 | 64.24 (22.82) | 93 | 61.87 (22.26) | 99 | 65.45 (22.08) |
| Arthritis Self-Efficacy | 88 | 63.56 (26.31) | 66 | 76.10 (20.97) | 69 | 76.19 (18.92) | 70 | 77.24 (20.97) | 131 | 62.39 (27.14) | 104 | 69.58 (25.02) | 93 | 68.08 (27.38) | 99 | 72.22 (24.84) |
