# Supplementary material for: Teens Taking Charge: A Randomized Controlled Trial of a Web-Based Self-Management Program With Telephone Support for Adolescents With Juvenile Idiopathic Arthritis
Source: J Med Internet Res. 2020 Jul 29;22(7):e16234. doi: 10.2196/16234 (PMC7424488; doi:10.2196/16234)
Supplement: Multimedia Appendix 5 [file jmir_v22i7e16234_app5.docx]

| **Outcome measure** | **Linear mixed model** | | |
| --- | --- | --- | --- |
|  | **Time** | **Condition** | **Time by condition** |
|  | **F value, *P*-value** | **F value, *P*-value** | **F value, *P*-value** |
| Anxiety | 0.65, >.99* | 0.00, <.99* | 1.60, >.99* |
| Depression | 1.30, >.99* | 1.79, >.99* | 1.55, >.99* |
| Adherence Report Questionnaire |  |  |  |
| Medications | 0.65, >.99* | 0.11, >.99* | 0.97, >.99* |
| Exercises | 1.01, >.99* | 0.39, .54* | 0.14, >.99* |
| Splints | 4.76, .28* | 3.21, .82* | 1.96, >.99* |
| Pain Coping Questionnaire | 1.12, >.99* | 1.14, >.99* | 2.09, >.99* |
| Medical Issues Questionnaire | 4.49, .096* | 0.47, >.99* | 2.53, .64* |
| Arthritis Self-Efficacy | 2.24, .86* | 7.69, .048* | 0.26, >.99* |
