# Supplementary material for: Teens Taking Charge: A Randomized Controlled Trial of a Web-Based Self-Management Program With Telephone Support for Adolescents With Juvenile Idiopathic Arthritis
Source: J Med Internet Res. 2020 Jul 29;22(7):e16234. doi: 10.2196/16234 (PMC7424488; doi:10.2196/16234)
Supplement: Multimedia Appendix 6 [file jmir_v22i7e16234_app6.docx]

| **Outcome** | **Teens Taking Charge intervention** | | | | | | | | **Education control** | | | | | | | | |
| --- | --- | --- | --- | --- | --- | --- | --- | --- | --- | --- | --- | --- | --- | --- | --- | --- | --- |
|  | **Baseline** | | **3 month** | | **6 month** | | **12 month** | | **Baseline** | | **3 month** | | **6 month** | | **12 month** | |  |
|  | **N** | **Mean (SD)** | **N** | **Mean (SD)** | **N** | **Mean (SD)** | **N** | **Mean (SD)** | **N** | **Mean (SD)** | **N** | **Mean (SD)** | **N** | **Mean (SD)** | **N** | **Mean (SD)** |  |
| Quality of life |  |  |  |  |  |  |  |  |  |  |  |  |  |  |  |  |  |
| Problems with pain | 75 | 38.17 (22.40) | 51 | 43.58 (22.15) | 63 | 45.22 (23.21) | 56 | 45.69 (21.21) | 122 | 39.57 (21.09) | 92 | 43.17 (21.03) | 87 | 40.30 (21.32) | 96 | 44.30 (21.89) |  |
| Problems with daily activities | 75 | 66.73 (13.67) | 51 | 66.47 (13.46) | 63 | 67.25 (13.64) | 56 | 68.50 (13.10) | 122 | 67.07 (14.05) | 92 | 67.51 (13.33) | 87 | 65.39 (15.10) | 96 | 66.11 (15.10) |  |
| Treatment problems | 75 | 50.53 (15.36) |  | 53.94 (15.73) | 63 | 57.69 (15.53) |  | 57.67 (14.39) | 122 | 50.54 (16.69) | 92 | 53.54 (16.16) | 87 | 53.04 (15.92) | 96 | 54.77  (17.21) |  |
| Worry | 75 | 48.71 (22.97) |  | 50.62 (22.46) | 63 | 52.46 (21.34) |  | 51.19 (23.66) | 122 | 41.11 (23.09) | 92 | 43.04 (26.15) | 87 | 41.86 (23.87) | 96 | 46.01 (25.66) |  |
| Communication problems | 75 | 51.80 (23.61) |  | 51.41 (21.76) | 63 | 55.26 (21.67) |  | 53.54 (21.74) | 122 | 47.24 (23.11) | 92 | 48.89 (24.04) | 87 | 47.68 (24.96) | 96 | 49.32 (23.49) |  |
| Adherence Report Questionnaire |  |  |  |  |  |  |  |  |  |  |  |  |  |  |  |  |  |
| Medications | 65 | 2.65 (2.89) | 38 | 3.26 (2.99) | 50 | 3.61 (3.29) | 42 | 3.42 (2.83) | 110 | 2.42 (2.76) | 75 | 2.74 (2.12) | 71 | 3.14 (2.33) | 74 | 3.34 (2.54) |  |
| Exercises | 48 | 4.07 (3.03) | 31 | 3.77 (2.48) | 41 | 4.27 (3.00) | 39 | 4.44 (2.48) | 86 | 3.73 (2.65) | 59 | 3.86 (2.62) | 51 | 4.42 (2.40) | 60 | 4.03 (2.62) |  |
| Splints | 11 | 1.36 (2.05)  11 | 5 | 4.50 (3.32)  5 | 5 | 1.80 (1.79)  5 | 9 | 2.78 (2.21)  9 | 9 | 3.17 (1.70)  9 | 8 | 2.00 (1.07)  8 | 7 | 2.93 (2.49) | 11 | 3.86 (3.01) |  |
| Arthritis Self-Efficacy | 72 | 75.40 (40.82) | 46 | 88.59 (44.47) | 57 | 81.74 (44.68) | 49 | 77.55 (39.41) | 118 | 80.34 (37.54) | 89 | 83.21 (43.46) | 84 | 81.31 (37.49) | 91 | 90.17 (39.45) |  |
| Medical Issues Questionnaire | 75 | 65.16 (22.83) | 51 | 72.72 (16.80) | 63 | 71.41 (20.22) | 56 | 72.06 (19.10) | 122 | 63.41 (22.10) | 92 | 67.20 (22.14) | 87 | 66.48 (20.99) | 95 | 71.56 (19.58) |  |
