# Supplementary material for: Teens Taking Charge: A Randomized Controlled Trial of a Web-Based Self-Management Program With Telephone Support for Adolescents With Juvenile Idiopathic Arthritis
Source: J Med Internet Res. 2020 Jul 29;22(7):e16234. doi: 10.2196/16234 (PMC7424488; doi:10.2196/16234)
Supplement: Multimedia Appendix 7 [file jmir_v22i7e16234_app7.docx]

| **Outcome measure** | **Linear mixed model** | | |
| --- | --- | --- | --- |
|  | **Time** | **Condition** | **Time by condition** |
|  | **F value, *P*-value** | **F value, *P*-value** | **F value, *P*-value** |
| Quality of life |  |  |  |
| Problems with pain | 2.20, .11 | 1.31, .25 | 1.61, .20 |
| Problems with daily activities | 0.78, .46 | 0.40, .53 | 0.91, .40 |
| Treatment problems | 2.62, .075 | 1.15, .28 | 1.24, .29 |
| Worry | 0.75, .47 | 0.94, .33 | 1.99, .14 |
| Communication problems | 0.79, .45 | 1.62, .20 | 0.84, .43 |
| Adherence Report Questionnaire |  |  |  |
| Medications | 1.67, >.99* | 1.31, >.99* | 0.11, >.99* |
| Exercises | 0.59, >.99* | 0.17, >.99* | 0.64, >.99* |
| Splints | 0.67, >.99* | 0.04, >.99* | 2.58, .75* |
| Arthritis Self-Efficacy | 0.58, >.99* | 0.02, >.99* | 3.47, .15* |
| Medical Issues Questionnaire | 3.07, .15* | 1.59, >.99* | 1.00, >.99* |

*p-values incorporate a Bonferroni adjustment for repeated testing
